# Treatment Gaps in Women Aged 65 Years and Older with Stress or Stress-Predominant Mixed Urinary Incontinence: Age-Stratified Patterns of Active Therapy, Containment Use, Frailty, and Anxiety

**DOI:** 10.3390/medicina62091778

**Published:** 2026-09-16

**Authors:** Doğucan Nuri Uğur, Kadir Böcü

**Affiliations:** Department of Urology, Niğde Ömer Halisdemir University Training and Research Hospital, Niğde 51200, Türkiye

**Keywords:** stress urinary incontinence, mixed urinary incontinence, older women, treatment gap, frailty, anxiety, pelvic floor muscle training

## Abstract

*Background and Objectives:* Older women with stress or stress-predominant mixed urinary incontinence may rely on pads or diapers despite bothersome symptoms. We examined age-stratified treatment patterns and whether age remained associated with a treatment gap after accounting for frailty and symptom burden. *Materials and Methods:* This single-center observational study combined record review (December 2023–April 2026) with a questionnaire assessment (19 June–3 August 2026). Within the 65–74, 75–84, and ≥85-year strata, 185, 182, and 183 women were screened; after 25, 22, and 23 exclusions or incomplete assessments, respectively, 160 evaluable women remained in each stratum. The treatment gap required International Consultation on Incontinence Questionnaire-Urinary Incontinence Short Form (ICIQ-UI SF) score ≥ 8, daily pad/diaper use, pad/diaper-only management, and no active treatment in the previous 12 months. Frailty and anxiety were assessed with the fatigue, resistance, ambulation, illnesses, and loss of weight (FRAIL) scale and Geriatric Anxiety Inventory. *Results:* Treatment-gap prevalence was 33.8%, 44.4%, and 55.0%. Supervised pelvic floor muscle training declined from 55.0% to 40.0% and 25.0%; recent active treatment from 50.0% to 35.0% and 20.0%; and previous continence surgery from 30.0% to 18.1% and 8.1%. After adjustment, age was not independently associated. Higher ICIQ-UI SF scores [adjusted odds ratio (aOR) 1.10 per point, 95% confidence interval (CI) 1.02–1.19] and stress-predominant mixed incontinence (aOR 1.53, 95% CI 1.03–2.25) remained associated. *Conclusions:* Containment dependence increased and active-treatment exposure decreased with age, but symptom burden and phenotype explained more adjusted risk than age. Regular reassessment may distinguish appropriate containment from remediable under-treatment.

## 1. Introduction

Urinary incontinence becomes more common as women age and is closely intertwined with multimorbidity, impaired mobility, functional dependence, and reduced quality of life [1,2,3,4]. Even so, many women delay seeking care or never receive a structured continence assessment. Symptoms may be accepted as an inevitable part of aging, concealed because of embarrassment, or managed privately when access to specialist services is limited [5,6].

Absorbent products are essential for comfort and dignity, particularly when active treatment is unsuitable, ineffective, or declined. They do not, however, treat the underlying continence disorder. Older adults often rely for long periods on pads, fluid restriction, and activity avoidance [7]. This may be entirely appropriate in some circumstances, but containment can also become the default response to a treatable condition. Conservative treatment remains relevant in later life: supervised pelvic floor muscle training (PFMT) can reduce leakage in older and frail people [8], and current European Association of Urology (EAU) guidance recommends at least three months of supervised intensive PFMT as first-line therapy for women with stress urinary incontinence (SUI) or mixed urinary incontinence (MUI), including older women [9].

Treatment decisions in older women cannot be based on age alone. Frailty, comorbidity, medication burden, cognition, mobility, caregiver support, treatment goals, and expected benefit all affect the feasibility of conservative and surgical options. Urinary incontinence is associated with frailty [10], and frailty influences both procedure selection and postoperative complications [11]. Surgical outcomes may also vary with age and health status, although many carefully selected older women still report meaningful improvement after continence surgery [12].

This study examined women aged 65 years or older with SUI or stress-predominant MUI. We compared age strata with respect to PFMT, medication use, previous continence surgery, containment dependence, frailty, and anxiety. We also tested whether chronological age remained associated with a prespecified treatment-gap outcome after adjustment for symptom severity and clinical complexity. We expected containment-only management to become more common with age, while anticipating that differences in symptom burden and health status would account for part of that gradient.

## 2. Materials and Methods

### 2.1. Study Design and Setting

This single-center observational analytical study combined a retrospective medical-record review with a one-time cross-sectional questionnaire assessment in the Department of Urology at Niğde Ömer Halisdemir University Training and Research Hospital, Niğde, Türkiye. The source frame comprised urology outpatient records in which clinical assessment had documented SUI or stress-predominant MUI during initial evaluation or follow-up. Reporting followed the Strengthening the Reporting of Observational Studies in Epidemiology (STROBE) recommendations.

The retrospective record period was December 2023–April 2026. The one-time questionnaire assessment was conducted from 19 June 2026 to 3 August 2026, after ethics approval had been granted on 17 June 2026.

### 2.2. Participants and Data Collection

**Figure 1:**
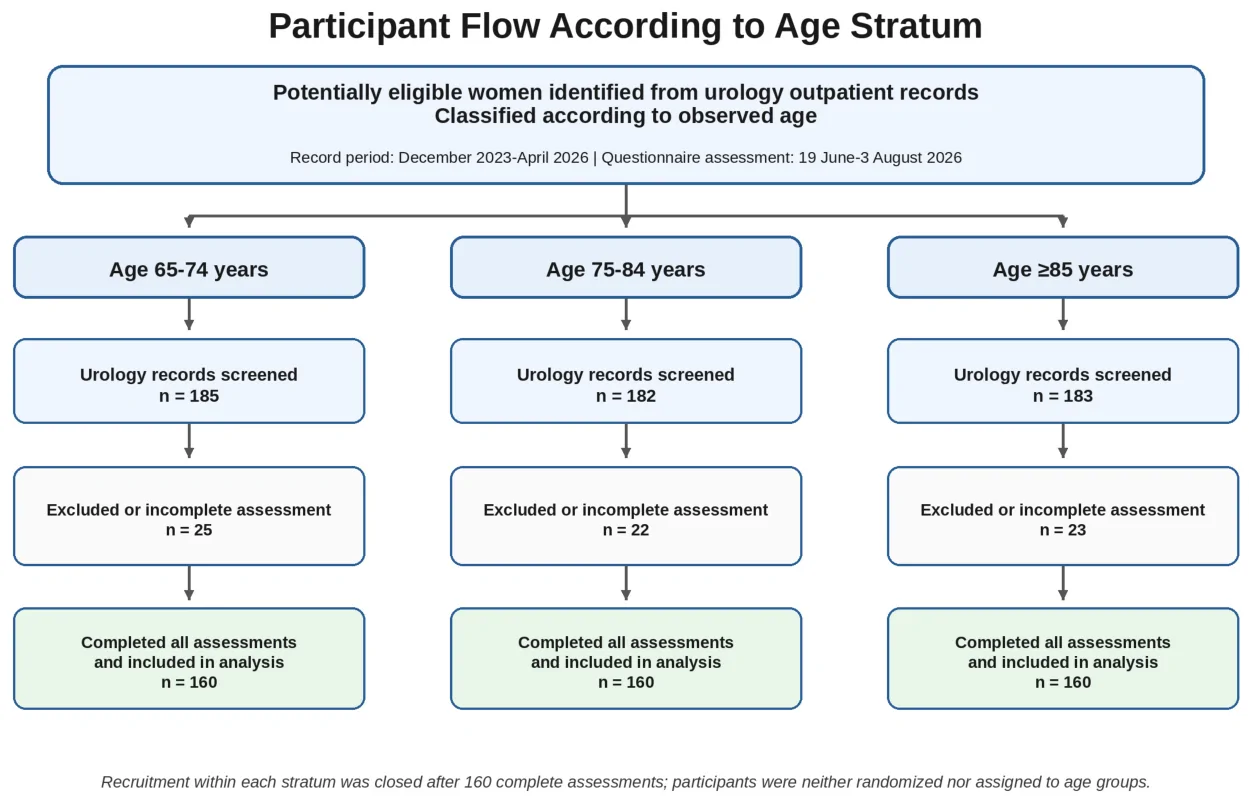
Participant flow according to age stratum. Potentially eligible women were classified according to their observed age and screened within the corresponding age stratum. In the 65–74-year stratum, 185 women were screened, 25 were excluded or had incomplete assessments, and 160 were included. In the 75–84-year stratum, 182 women were screened, 22 were excluded or had incomplete assessments, and 160 were included. In the ≥85-year stratum, 183 women were screened, 23 were excluded or had incomplete assessments, and 160 were included. Recruitment within each stratum was closed after 160 complete assessments had been obtained. Participants were not assigned to age groups.

Medical records of women aged 65 years or older were screened when clinical assessment had identified SUI or stress-predominant MUI. To permit direct comparisons with similar statistical precision across the three age strata, a prespecified balanced recruitment target of 160 complete assessments per stratum was used. Potentially eligible women identified from the source urology records were classified according to their observed age as 65–74, 75–84, or ≥85 years. Within each age stratum, eligible women were ordered chronologically according to the date of the qualifying urology record and were approached consecutively, without selective omission. Recruitment for a stratum was closed once 160 women had completed all required study assessments. In total, 185, 182, and 183 women were screened in the 65–74, 75–84, and ≥85-year strata, respectively. Following 25, 22, and 23 documented exclusions or incomplete assessments, 160 evaluable participants remained in each stratum, yielding a total analytic cohort of 480 women. Participant screening, exclusions, and inclusion by age stratum are summarized in Figure 1. Across all strata, the 70 exclusions or incomplete assessments comprised incomplete International Consultation on Incontinence Questionnaire-Urinary Incontinence Short Form (ICIQ-UI SF) or Geriatric Anxiety Inventory (GAI) data (*n* = 25), a non-eligible incontinence phenotype (*n* = 18), neurogenic lower urinary tract dysfunction (*n* = 10), severe cognitive or communication limitation preventing valid assessment (*n* = 9), and withdrawal or duplicate records (*n* = 8). Participants were not randomized or assigned to age groups, and no selection was based on the study outcomes. Because the equal stratum sizes were an intentional design feature, the cohort was not intended to reproduce the natural age distribution of the clinic population.

Following ethics approval, eligible women who could complete the study measures were invited to the one-time assessment. Trained study personnel from the Department of Urology administered the Turkish-language instruments in a standardized format. Participants provided their own responses; when assistance was needed, items were read verbatim and the participant’s answer was recorded without interpretation. Proxy completion was not accepted. The retrospective record review provided information on comorbidities, regular medication use, previous continence surgery, prior medical and conservative treatment, and pad or diaper use. The one-time assessment provided current symptom severity, frailty, anxiety, functional status, and patient-reported care information. Reasons such as medical/frailty concern, previous treatment refusal, access or caregiver barriers, and previous treatment failure referred to barriers to sustained active treatment rather than inability to complete a single assessment; women unable to provide valid responses because of severe cognitive or communication limitation were excluded.

Age was categorized a priori as 65–74, 75–84, and ≥85 years. No upper age limit was imposed; the ≥85-year category was an open-ended oldest-old stratum rather than a 15-year interval. The mean age in this stratum was 90.4 ± 3.4 years (Table 1).

Urodynamics was not a protocol-mandated or standardized study procedure, and no cohort-level urodynamic variable was collected. Eligibility, phenotype classification, and treatment-gap derivation were therefore based on symptom pattern, clinical assessment, and cough stress-test findings rather than urodynamic results. Urodynamic testing may have been performed selectively when clinically indicated in routine care, but those findings were not part of the analytical dataset. This approach is consistent with current guidance, which does not recommend routine urodynamics before treatment of uncomplicated, clinically demonstrable SUI and reserves testing for diagnostic uncertainty or situations in which the findings may alter invasive treatment decisions [9].

Recorded demographic and geriatric variables included age, body mass index, parity, education, living arrangement, caregiver support, mobility-aid use, dependence in activities of daily living (ADL), numbers of chronic conditions and regular medications, and falls during the preceding 12 months. Polypharmacy was defined as the regular use of five or more medications.

### 2.3. Incontinence, Frailty, and Anxiety Measures

Incontinence frequency, amount, and effect on daily life were measured with the International Consultation on Incontinence Questionnaire-Urinary Incontinence Short Form (ICIQ-UI SF) [13]. Total scores range from 0 to 21 and were categorized as mild (1–5), moderate (6–12), severe (13–18), or very severe (19–21) [14]. The ICIQ-UI SF was used to quantify symptom burden rather than to determine treatment eligibility. Incontinence phenotype was assigned from the symptom pattern and clinical assessment. The dataset also included symptom duration, cough stress-test findings, daily and nighttime containment use, diaper use, previous care-seeking, and limitation of social activity.

Frailty was assessed with the five-item FRAIL scale, which covers fatigue, resistance, ambulation, illnesses, and weight loss [15,16]. Scores of 0, 1–2, and 3–5 were categorized as robust, pre-frail, and frail, respectively.

Anxiety was assessed with the 20-item Geriatric Anxiety Inventory (GAI) [17] using the validated Turkish version [18]. A score ≥ 9 was considered a positive screening result. The GAI was used as a measure of anxiety burden and not as a psychiatric diagnosis.

### 2.4. Treatment Variables and Primary Outcome

Treatment variables comprised documented active treatment ever offered, previous supervised PFMT, urgency-directed medication, duloxetine exposure in selected patients, previous continence surgery, active treatment during the preceding 12 months, current management, interest in surgery, and clinical surgical eligibility. “Active treatment” was defined as clinician-directed management intended to improve urinary incontinence: supervised PFMT; pharmacotherapy appropriate to the stress or urgency component; a continence procedure or operation; structured postoperative continence follow-up; or a combination of these. Pads or diapers alone, observation without active intervention, and self-directed exercise or lifestyle measures without a supervised program were not classified as active treatment. “Active treatment ever offered” and “active treatment received during the preceding 12 months” were overlapping binary variables with different time frames and were not mutually exclusive. Previous continence surgery was recorded as a binary historical exposure; procedure type, number of procedures, and standardized outcomes were not sufficiently complete for reliable cohort-level reporting. Duloxetine was analyzed only as selected-patient historical exposure and was not presented as universal or first-line treatment.

The primary treatment-gap outcome was defined a priori as the simultaneous presence of an ICIQ-UI SF score ≥ 8, use of at least one pad or diaper per day, current management with pads or diapers alone, and no active treatment during the preceding 12 months. Thus, ongoing pad use did not constitute a treatment gap when it accompanied active therapy. The ≥8 cutoff was a study-specific operational threshold rather than an established ICIQ-UI SF category boundary or guideline-mandated treatment threshold; it was selected to exclude the lowest part of the moderate range without restricting the outcome to severe symptoms only. Sensitivity analyses repeated the complete definition using the established category boundaries of ≥6 and ≥13. The corresponding treatment-gap rates were 35.0%, 45.0%, and 55.0% at ≥6 and 13.8%, 28.8%, and 40.6% at ≥13 across the 65–74, 75–84, and ≥85-year strata, respectively.

### 2.5. Ethics

The study was conducted in accordance with the Declaration of Helsinki and was approved by the Niğde Ömer Halisdemir University Faculty of Medicine Non-Interventional Clinical Research Ethics Committee (protocol code 2026/107; decision no. 2026/108; approval date 17 June 2026).

Informed consent: Written informed consent was obtained from all participants before the one-time questionnaire assessment.

### 2.6. Statistical Analysis

Continuous and ordinal variables are reported as mean ± standard deviation or median [interquartile range], and categorical variables as number (percentage). Age-group comparisons used the Kruskal–Wallis test for continuous or ordinal variables and the Pearson chi-square test for categorical variables. No values were imputed. Two-sided *p* values < 0.05 were considered statistically significant; because several secondary comparisons were exploratory, their *p* values were interpreted without claiming confirmatory multiplicity control.

The primary multivariable logistic regression model examined the treatment gap and included age group, FRAIL score, ICIQ-UI SF total score, incontinence phenotype, comorbidity count, polypharmacy, any ADL dependence, living arrangement, and caregiver support. A second logistic model examined a positive anxiety screen and included age group, ICIQ-UI SF score, incontinence phenotype, daily pad or diaper count, nighttime containment, FRAIL score, and social-activity limitation. Results are reported as adjusted odds ratios (aORs) with 95% confidence intervals (CIs). Discrimination is summarized using the area under the receiver operating characteristic curve (AUC). Analyses were performed with Python 3.13.5, SciPy 1.17.0, statsmodels 0.14.6, and scikit-learn 1.8.0.

## 3. Results

### 3.1. Participant and Geriatric Characteristics

In the 65–74-year stratum, 185 women were screened, 25 were excluded or had incomplete assessments, and 160 were included. In the 75–84-year stratum, 182 women were screened, 22 were excluded or had incomplete assessments, and 160 were included; the corresponding numbers in the ≥85-year stratum were 183, 23, and 160. The final analytic cohort comprised 480 women. Across all strata, the 70 exclusions or incomplete assessments comprised incomplete ICIQ-UI SF or GAI data (*n* = 25), a non-eligible incontinence phenotype (*n* = 18), neurogenic lower urinary tract dysfunction (*n* = 10), severe cognitive or communication limitation (*n* = 9), and withdrawal or duplicate records (*n* = 8). Mean age was 69.3 ± 2.8 years in the 65–74-year stratum, 79.5 ± 2.8 years in the 75–84-year stratum, and 90.4 ± 3.4 years in the open-ended ≥85-year stratum (*p* < 0.001).

Body mass index was similar across age strata (median 28.8, 29.0, and 29.3 kg/m^2^; *p* = 0.204), whereas median comorbidity count increased from 2 to 3 and 4 and median medication count from 4 to 5 and 7 (both *p* < 0.001). Polypharmacy was present in 44/160 (27.5%), 97/160 (60.6%), and 146/160 (91.2%) women, respectively (*p* < 0.001).

Functional and frailty burden also increased with age. Any ADL dependence was recorded in 32/160 (20.0%), 44/160 (27.5%), and 77/160 (48.1%); caregiver support in 43/160 (26.9%), 63/160 (39.4%), and 130/160 (81.2%); and mobility-aid use in 36/160 (22.5%), 61/160 (38.1%), and 88/160 (55.0%) women (all *p* < 0.001). Frailty was present in 16/160 (10.0%), 40/160 (25.0%), and 88/160 (55.0%) women, respectively (*p* < 0.001) (Table 1).

### 3.2. Incontinence and Anxiety Burden

Stress-predominant MUI became more common across age strata, affecting 56/160 (35.0%), 72/160 (45.0%), and 88/160 (55.0%) women (*p* = 0.002). Median ICIQ-UI SF scores were 11 [9–13], 13 [10–14], and 14 [12–16], while median symptom duration was 56.0 [31.2–73.0], 70.0 [53.8–87.5], and 88.5 [68.8–113.5] months, respectively (both *p* < 0.001). Median daily containment use increased from 2 [2–3] to 3 [2–4] and 4 [3–5] products (*p* < 0.001). Nighttime containment was reported by 64/160 (40.0%), 88/160 (55.0%), and 112/160 (70.0%), and diaper use by 12/160 (7.5%), 38/160 (23.8%), and 74/160 (46.2%) women (both *p* < 0.001) (Table 2).

Positive anxiety-screen rates were 40/160 (25.0%), 48/160 (30.0%), and 45/160 (28.1%) and did not differ significantly across age strata (*p* = 0.601), although median GAI scores increased from 4.0 [3.0–7.8] to 5.0 [3.0–11.0] and 6.0 [4.0–12.0] (*p* < 0.001). Moderate or severe limitation of social activity was reported by 88/160 (55.0%), 90/160 (56.2%), and 102/160 (63.8%) participants (*p* = 0.229).

### 3.3. Treatment Patterns and the Treatment Gap

**Figure 2:**
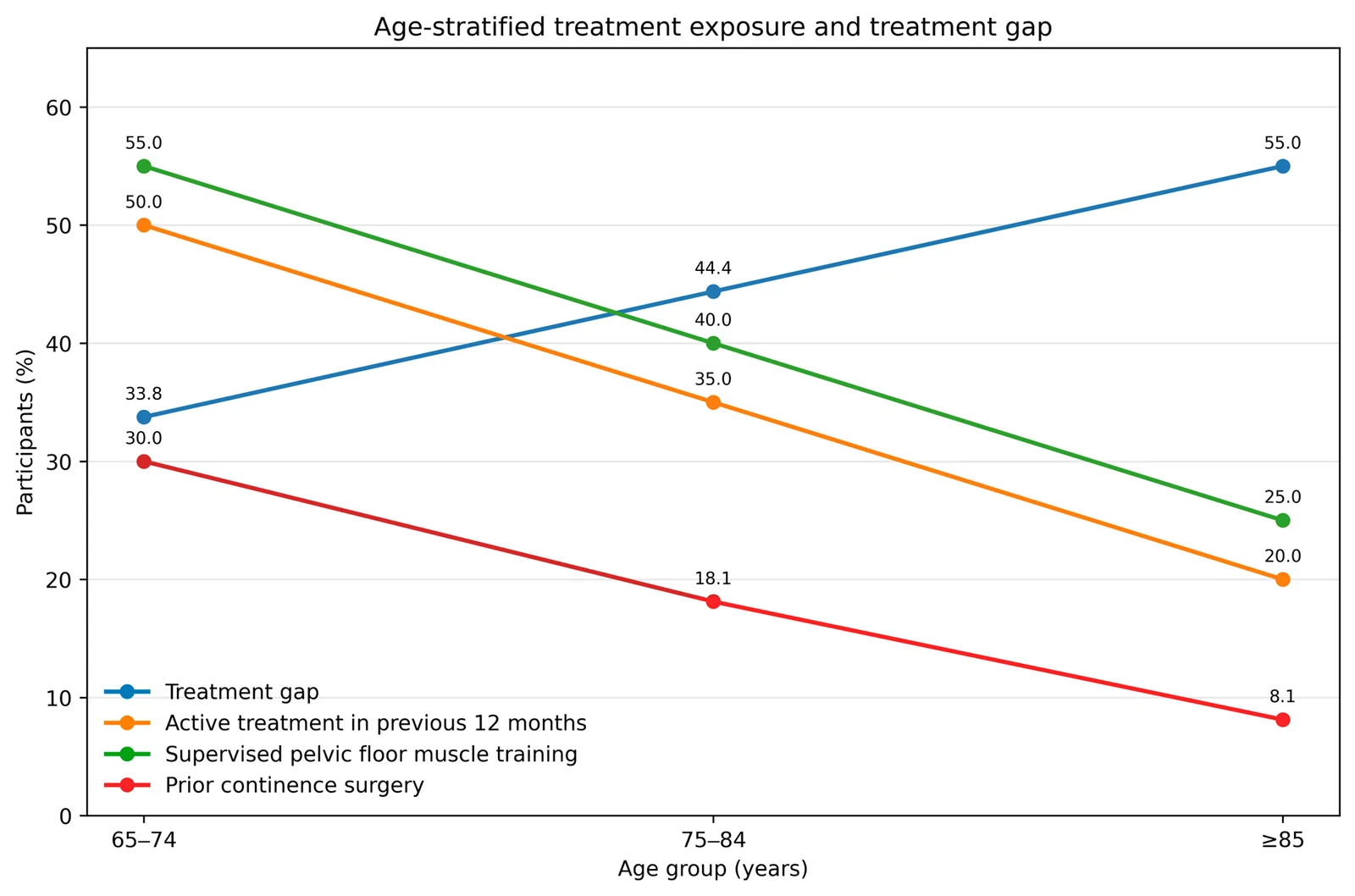
Age-stratified treatment exposure and treatment gap. PFMT, pelvic floor muscle training.

Exposure to clinician-directed continence treatment decreased with age. Documented active treatment had ever been offered to 120/160 (75.0%), 96/160 (60.0%), and 72/160 (45.0%) women; supervised PFMT had been used by 88/160 (55.0%), 64/160 (40.0%), and 40/160 (25.0%); and active treatment during the preceding 12 months had been received by 80/160 (50.0%), 56/160 (35.0%), and 32/160 (20.0%), respectively (all *p* < 0.001). Previous continence surgery was documented in 90 women overall: 48/160 (30.0%), 29/160 (18.1%), and 13/160 (8.1%) across the three strata (*p* < 0.001). In parallel, pad/diaper-only management increased from 56/160 (35.0%) to 72/160 (45.0%) and 88/160 (55.0%) (*p* = 0.002) (Table 3 and Figure 2).

The primary treatment gap was present in 54/160 (33.8%) women aged 65–74 years, 71/160 (44.4%) aged 75–84 years, and 88/160 (55.0%) aged ≥85 years (*p* < 0.001). Among the 213 women who met the definition, the recorded reason for no active treatment was treatment never offered in 73 (34.3%), medical or frailty concern in 50 (23.5%), patient refusal in 40 (18.8%), an access or caregiver barrier in 33 (15.5%), and previous treatment failure in 17 (8.0%). These categories described barriers to receiving or sustaining active treatment and did not indicate inability to complete the one-time assessment. The age gradient remained when the ICIQ-UI SF threshold was changed to ≥6 (35.0%, 45.0%, and 55.0%) or ≥13 (13.8%, 28.8%, and 40.6%).

The primary treatment gap required ICIQ-UI SF ≥ 8, at least one pad or diaper daily, pad/diaper-only current management, and no active treatment during the preceding 12 months. Active treatment comprised clinician-directed supervised PFMT, phenotype-appropriate pharmacotherapy, a continence procedure or operation, structured postoperative continence follow-up, or a combination of these. The lifetime “ever offered” and preceding-12-month treatment indicators overlap and must not be added. Previous continence surgery was available as a binary historical exposure; procedure type, repeat procedures, and standardized outcomes were not sufficiently complete for reliable analysis. Duloxetine denotes selected-patient historical exposure and is not presented as universal or first-line therapy. PFMT, pelvic floor muscle training.

### 3.4. Multivariable Models

In a model containing age stratum alone, the odds of the treatment gap were higher at ages 75–84 years (OR 1.57, 95% CI 1.00–2.46; *p* = 0.052) and ≥85 years (OR 2.40, 95% CI 1.53–3.77; *p* < 0.001) than at ages 65–74 years. After multivariable adjustment, neither age 75–84 years (aOR 1.12, 95% CI 0.66–1.88; *p* = 0.679) nor age ≥ 85 years (aOR 1.09, 95% CI 0.55–2.15; *p* = 0.803) remained independently associated. Higher ICIQ-UI SF scores (aOR 1.10 per point, 95% CI 1.02–1.19; *p* = 0.014) and stress-predominant MUI (aOR 1.53, 95% CI 1.03–2.25; *p* = 0.033) remained associated with the treatment gap. The model AUC was 0.669 (Table 4).

In the anxiety model, higher ICIQ-UI SF scores (aOR 1.71 per point, 95% CI 1.46–2.01; *p* < 0.001), stress-predominant MUI (aOR 1.83, 95% CI 1.08–3.10; *p* = 0.025), nighttime containment (aOR 2.72, 95% CI 1.54–4.79; *p* < 0.001), and each higher category of social-activity limitation (aOR 2.63, 95% CI 1.99–3.48; *p* < 0.001) were independently associated with a positive screen. Daily pad/diaper count and FRAIL score were not independently associated. Adjusted age coefficients were inverse despite similar unadjusted screening rates: aOR 0.49 (95% CI 0.24–1.00; *p* = 0.049) for ages 75–84 years and aOR 0.11 (95% CI 0.05–0.28; *p* < 0.001) for ages ≥ 85 years. These estimates should not be interpreted as a protective effect of advanced age; adaptation, differences in symptom reporting, selective participation, and residual confounding are plausible explanations. The model AUC was 0.878.

## 4. Discussion

The descriptive pattern was consistent across several measures: women in older age strata were more likely to depend on pads or diapers and less likely to have received supervised PFMT, recent active treatment, or previous continence surgery. Treatment-gap prevalence increased from 33.8% at ages 65–74 years to 55.0% at ages ≥ 85 years. Once symptom severity, incontinence phenotype, frailty, comorbidity, medication burden, functional dependence, living arrangement, and caregiver support were considered together, chronological age no longer explained the treatment gap independently. The crude age gradient therefore appears to reflect both greater clinical burden and differences in how care is offered, accessed, or accepted.

The distinction between containment and treatment is central to interpretation. Pads and diapers are often appropriate and should not be framed as therapeutic failure. They may be the preferred option for a woman with substantial comorbidity, previous treatment failure, limited treatment goals, or a well-informed decision to avoid further intervention. The study definition therefore counted containment as a gap only when clinically relevant leakage, daily product use, pad/diaper-only management, and absence of active treatment were all present. Even with this conservative definition, 73/213 (34.3%) gap cases had no record that active treatment had ever been offered, and 33/213 (15.5%) had an access or caregiver barrier. These are potentially modifiable points in the continence-care pathway.

The fall in supervised PFMT exposure—from 88/160 (55.0%) in the youngest stratum to 40/160 (25.0%) in the oldest—is clinically important. Conservative treatment can benefit older and frail people [8], and the EAU recommends supervised intensive PFMT for at least three months as first-line treatment for women with SUI or MUI, including older women [9]. Frailty, poor mobility, and dependence may require shorter sessions, home-based or remote delivery, caregiver participation, or closer supervision, but they do not automatically remove the potential value of treatment.

Frailty rose from 10.0% to 55.0% across age strata, yet the FRAIL score was not independently associated with the operational treatment-gap outcome. This does not mean that frailty is unimportant. Frailty is associated with urinary incontinence [10], affects procedure selection, and is linked to complications after procedure-based treatment [11]. In the present analysis, symptom severity and stress-predominant MUI were more directly related to the treatment-gap endpoint, which combined potentially remediable under-treatment with clinically appropriate decisions not to intervene. More detailed assessment of physiological reserve, cognition, goals, treatment preferences, and access barriers would help separate these pathways.

Previous continence surgery also became less common with age, declining from 48/160 (30.0%) to 29/160 (18.1%) and 13/160 (8.1%). Lower surgical use should not automatically be interpreted as inequity: operative risk, expected benefit, patient preference, and the relative importance of stress and urgency symptoms all influence decision-making. At the same time, chronological age should not serve as an exclusion criterion by itself. Register data indicate that health status and age influence outcomes, but meaningful improvement remains possible in selected older women [12]. Because the historical extraction recorded previous surgery as a binary variable, the present study cannot compare specific procedures, repeat operations, or standardized postoperative outcomes. The appropriate response is individualized counseling rather than a fixed age threshold.

Anxiety showed a different pattern. Unadjusted positive-screen rates were similar across age strata (25.0%, 30.0%, and 28.1%), while symptom severity, stress-predominant MUI, nighttime containment, and social limitation were strongly associated with anxiety in the adjusted model. Previous work has likewise shown that severity may matter more than subtype for quality of life [19], although MUI can impose a particularly broad burden [20]. The inverse adjusted coefficients for older age strata should be viewed cautiously. They may reflect adaptation, different expectations, selective participation, reporting differences, or unmeasured psychological and social factors; they do not establish that advanced age protects against anxiety.

Recent literature reinforces both the persistence of urinary incontinence in older women and the need for accessible, individualized care. Contemporary reviews describe continued under-recognition during later-life health-care encounters [21], limited but evolving evidence for home-based interventions [22], and the feasibility of online group PFMT and telerehabilitation for older women [23,24]. Newer work also emphasizes careful interpretation of frailty instruments in women with geriatric urinary incontinence [25]. A 2025 network meta-analysis found that evidence comparing treatments specifically in older women remains limited and often low-certainty [26], while a recent collaborative review highlights the continuing gap between SUI prevalence, diagnosis, and treatment uptake [27]. These studies support expanding access and assessment while avoiding the assumption that any single treatment is appropriate for every older woman.

The findings support a practical two-step approach. First, older women using absorbent products should be asked whether containment is an informed choice, a complement to treatment, or a substitute for care that has never been offered or cannot be accessed. Second, treatment planning should combine symptom severity and phenotype with frailty, mobility, cognition, goals, caregiver resources, and likely treatment benefit. This approach preserves the value of containment while reducing the risk that it becomes the only long-term response to a treatable problem.

This study has several limitations. It was conducted at one center, and the combination of retrospective treatment-history data with a one-time cross-sectional assessment does not permit temporal or causal conclusions. Historical treatment information may have been incomplete or inconsistently documented, and participant-reported histories may be affected by recall. The prespecified balanced recruitment target and stratum-specific stopping rule yielded equal age-stratum sizes, improving between-stratum precision but not reproducing the natural age distribution of the clinic population; pooled proportions should therefore not be interpreted as population prevalence estimates. The ≥85-year category was open-ended, and only its mean and standard deviation, rather than a year-by-year distribution, were available for reporting. The ICIQ-UI SF ≥ 8 threshold was a study-specific operational cutoff rather than a validated or guideline-mandated treatment threshold. Urodynamics was not standardized or included in the analytical dataset, so the study does not provide urodynamic phenotyping. Previous continence surgery was available only as a binary historical variable, precluding procedure-specific and outcome-specific analyses. Detailed treatment intensity, adherence, patient goals, socioeconomic and transportation factors, clinician-level factors, and longitudinal outcomes were not captured. Anxiety was evaluated with a screening instrument rather than a diagnostic interview. Multiple secondary comparisons were made without multiplicity adjustment, and both regression models were evaluated in the same dataset in which they were developed; external validation was not performed. Nevertheless, the large balanced cohort, complete primary-outcome data, explicit distinction between containment and active treatment, and simultaneous assessment of frailty, symptom burden, treatment exposure, and anxiety provide a clinically useful view of continence-care pathways in later life.

## 5. Conclusions

Older women showed progressively greater dependence on pads and diapers and lower exposure to supervised PFMT, recent active treatment, and previous continence surgery. The age gradient in the treatment gap was not independent after adjustment; symptom severity and stress-predominant MUI were more informative. Continence care in later life should assess whether containment reflects an informed, appropriate choice or an unresolved barrier to active treatment, while incorporating frailty, function, preferences, and caregiver resources into shared decision-making.

## Figures and Tables

**Table 1:** Demographic and geriatric characteristics by age group.

Characteristic	65–74 Years (*n* = 160)	75–84 Years (*n* = 160)	≥85 Years (*n* = 160)	*p*
Participants, *n*	160	160	160	-
Age, years	69.3 ± 2.8	79.5 ± 2.8	90.4 ± 3.4	<0.001
Body mass index, kg/m^2^	28.8 [25.9–31.8]	29.0 [25.4–31.5]	29.3 [27.0–32.4]	0.204
Parity	3 [2–4]	4 [2–4]	3 [3–4]	0.269
Comorbidity count	2 [1–3]	3 [2–4]	4 [3–5]	<0.001
Medication count	4 [2–5]	5 [4–6]	7 [6–8]	<0.001
Polypharmacy, *n* (%)	44 (27.5)	97 (60.6)	146 (91.2)	<0.001
Any ADL dependence, *n* (%)	32 (20.0)	44 (27.5)	77 (48.1)	<0.001
Caregiver support, *n* (%)	43 (26.9)	63 (39.4)	130 (81.2)	<0.001
Any mobility aid, *n* (%)	36 (22.5)	61 (38.1)	88 (55.0)	<0.001
FRAIL score	0 [0–1]	1 [0–2]	3 [1–3]	<0.001
Robust, *n* (%)	88 (55.0)	48 (30.0)	16 (10.0)	<0.001
Pre-frail, *n* (%)	56 (35.0)	72 (45.0)	56 (35.0)	
Frail, *n* (%)	16 (10.0)	40 (25.0)	88 (55.0)	
Falls in preceding 12 months	0 [0–1]	1 [0–1]	1 [0–1]	<0.001

Values are mean ± standard deviation, median [interquartile range], or *n* (%). Abbreviations: activities of daily living (ADL); fatigue, resistance, ambulation, illnesses, and loss of weight (FRAIL).

**Table 2:** Incontinence severity, containment use, and anxiety burden.

Characteristic	65–74 Years (*n* = 160)	75–84 Years (*n* = 160)	≥85 Years (*n* = 160)	*p*
Stress-predominant mixed UI, *n* (%)	56 (35.0)	72 (45.0)	88 (55.0)	0.002
Symptom duration, months	56.0 [31.2–73.0]	70.0 [53.8–87.5]	88.5 [68.8–113.5]	<0.001
ICIQ-UI SF total score	11 [9–13]	13 [10–14]	14 [12–16]	<0.001
Mild, *n* (%)	1 (0.6)	0 (0.0)	0 (0.0)	<0.001
Moderate, *n* (%)	113 (70.6)	74 (46.2)	43 (26.9)	
Severe, *n* (%)	46 (28.8)	84 (52.5)	106 (66.2)	
Very severe, *n* (%)	0 (0.0)	2 (1.2)	11 (6.9)	
Positive cough stress test, *n* (%)	148 (92.5)	143 (89.4)	142 (88.8)	0.481
Pads/diapers per day	2 [2–3]	3 [2–4]	4 [3–5]	<0.001
Nighttime pad/diaper use, *n* (%)	64 (40.0)	88 (55.0)	112 (70.0)	<0.001
Diaper use, *n* (%)	12 (7.5)	38 (23.8)	74 (46.2)	<0.001
GAI total score	4.0 [3.0–7.8]	5.0 [3.0–11.0]	6.0 [4.0–12.0]	<0.001
Positive anxiety screen, *n* (%)	40 (25.0)	48 (30.0)	45 (28.1)	0.601
Moderate/severe social limitation, *n* (%)	88 (55.0)	90 (56.2)	102 (63.8)	0.229

Values are median [interquartile range] or *n* (%). GAI, Geriatric Anxiety Inventory; ICIQ-UI SF, International Consultation on Incontinence Questionnaire-Urinary Incontinence Short Form; UI, urinary incontinence.

**Table 3:** Age-stratified treatment patterns and treatment gap.

Characteristic	65–74 Years (*n* = 160)	75–84 Years (*n* = 160)	≥85 Years (*n* = 160)	*p*
Ever sought professional care, *n* (%)	145 (90.6)	131 (81.9)	134 (83.8)	0.065
Documented active treatment ever offered (lifetime), *n* (%)	120 (75.0)	96 (60.0)	72 (45.0)	<0.001
Supervised PFMT ever, *n* (%)	88 (55.0)	64 (40.0)	40 (25.0)	<0.001
Urgency-directed medication ever, *n* (%)	34 (21.2)	44 (27.5)	42 (26.2)	0.393
Current urgency-directed medication, *n* (%)	22 (13.8)	24 (15.0)	16 (10.0)	0.382
Duloxetine exposure ever (selected patients), *n* (%)	19 (11.9)	15 (9.4)	11 (6.9)	0.308
Previous continence surgery (binary historical variable), *n* (%)	48 (30.0)	29 (18.1)	13 (8.1)	<0.001
Received active treatment during the preceding 12 months, *n* (%)	80 (50.0)	56 (35.0)	32 (20.0)	<0.001
Current pad/diaper-only management, *n* (%)	56 (35.0)	72 (45.0)	88 (55.0)	0.002
Primary treatment gap, *n* (%)	54 (33.8)	71 (44.4)	88 (55.0)	<0.001
Interested in surgery, *n* (%)	84 (52.5)	55 (34.4)	33 (20.6)	<0.001
Clinically eligible for surgery, *n* (%)	94 (58.8)	91 (56.9)	58 (36.2)	<0.001

**Table 4:** Multivariable logistic regression models.

Predictor	Adjusted Odds Ratio	95% CI	*p*
Panel A. Primary treatment-gap model			
Age 75–84 years (reference: 65–74)	1.12	0.66–1.88	0.679
Age ≥ 85 years (reference: 65–74)	1.09	0.55–2.15	0.803
FRAIL score, per point	1.08	0.90–1.29	0.390
ICIQ-UI SF score, per point	1.10	1.02–1.19	0.014
Stress-predominant mixed vs. stress UI	1.53	1.03–2.25	0.033
Comorbidity count, per condition	1.15	0.97–1.35	0.109
Polypharmacy	1.01	0.64–1.61	0.965
Any ADL dependence	1.33	0.84–2.08	0.220
Living alone	0.71	0.44–1.17	0.177
Residential care	0.86	0.39–1.91	0.715
Caregiver support	0.95	0.59–1.53	0.841
Panel B. Positive anxiety-screen model			
Age 75–84 years (reference: 65–74)	0.49	0.24–1.00	0.049
Age ≥ 85 years (reference: 65–74)	0.11	0.05–0.28	<0.001
ICIQ-UI SF score, per point	1.71	1.46–2.01	<0.001
Stress-predominant mixed vs. stress UI	1.83	1.08–3.10	0.025
Pads/diapers per day	1.15	0.83–1.59	0.400
Nighttime pad/diaper use	2.72	1.54–4.79	<0.001
FRAIL score, per point	0.85	0.70–1.04	0.112
Social-activity limitation, per category	2.63	1.99–3.48	<0.001

Panel A: *n* = 480, 213 treatment-gap events, AUC = 0.669. Panel B: *n* = 480, 133 positive anxiety screens, AUC = 0.878. Reference phenotype: stress urinary incontinence. Reference living arrangement in Panel A: living with a spouse or family. ADL, activities of daily living; CI, confidence interval; MUI, mixed urinary incontinence; UI, urinary incontinence.

## Data Availability

The participant-level data are not publicly available because they contain clinical information subject to institutional, ethical, and privacy restrictions. De-identified data may be made available by the corresponding author upon reasonable request and after the required institutional and ethics approvals.

## Abbreviations

ADL, activities of daily living; AUC, area under the receiver operating characteristic curve; CI, confidence interval; GAI, Geriatric Anxiety Inventory; ICIQ-UI SF, International Consultation on Incontinence Questionnaire-Urinary Incontinence Short Form; MUI, mixed urinary incontinence; PFMT, pelvic floor muscle training; SUI, stress urinary incontinence; UI, urinary incontinence.
